# AutoEdge-CCP: A novel approach for predicting cancer-associated circRNAs and drugs based on automated edge embedding

**DOI:** 10.1371/journal.pcbi.1011851

**Published:** 2024-01-30

**Authors:** Yaojia Chen, Jiacheng Wang, Chunyu Wang, Quan Zou

**Affiliations:** 1 Institute of Fundamental and Frontier Sciences, University of Electronic Science and Technology of China, Chengdu, China; 2 Yangtze Delta Region Institute (Quzhou), University of Electronic Science and Technology of China, Quzhou, China; 3 Faculty of Computing, Harbin Institute of Technology, Harbin, China; Pacific Lutheran University, UNITED STATES

## Abstract

The unique expression patterns of circRNAs linked to the advancement and prognosis of cancer underscore their considerable potential as valuable biomarkers. Repurposing existing drugs for new indications can significantly reduce the cost of cancer treatment. Computational prediction of circRNA-cancer and drug-cancer relationships is crucial for precise cancer therapy. However, prior computational methods fail to analyze the interaction between circRNAs, drugs, and cancer at the systematic level. It is essential to propose a method that uncover more valuable information for achieving cancer-centered multi-association prediction. In this paper, we present a novel computational method, AutoEdge-CCP, to unveil cancer-associated circRNAs and drugs. We abstract the complex relationships between circRNAs, drugs, and cancer into a multi-source heterogeneous network. In this network, each molecule is represented by two types information, one is the intrinsic attribute information of molecular features, and the other is the link information explicitly modeled by autoGNN, which searches information from both intra-layer and inter-layer of message passing neural network. The significant performance on multi-scenario applications and case studies establishes AutoEdge-CCP as a potent and promising association prediction tool.

## Author summary

CircRNAs serve as crucial biomarkers and drug targets in cancer therapy. Predicting cancer-associated circRNAs and drugs contributes to uncover intricate molecular mechanisms driving tumorigenesis, thus offering novel insights into cancer diagnosis, treatment, and research. However, prevailing predictive methods often neglect the comprehensive interactions within circRNAs, drugs, and cancer, leading to an incomplete understanding of their complex interplay. In response, we introduce AutoEdge-CCP, a framework that models circRNA-cancer-drug interactions within a multi-source heterogeneous network. Each molecule combines intrinsic attribute information describing molecular features with interaction information derived through autoGNN, revealing pivotal circRNAs and drugs associated with cancer. Experimental results across multi-scenario attest to AutoEdge-CCP’s superior performance compared to competing methods, particularly in predicting novel circRNAs and drugs associated with cancer. Additionally, visualization of edge embeddings and case studies provide interpretable insights into the prediction outcomes.

## Introduction

Cancer is a profoundly intricate disease characterized by a diverse array of mutations occurring within the genome, transcriptome, and proteome [1]. Most transcriptomic investigations have primarily concentrated on the dynamic changes in linear transcripts during cancer initiation and progression. Regrettably, these studies have often overlooked circular RNAs (circRNAs), that are formed by RNA polymerase II transcription and covalent back-splicing to form a closed circular structure [2]. Differential analysis of circRNA expression profiles in various tumor tissues and adjacent normal tissues has revealed that some circRNAs are upregulated or downregulated in tumors, thereby promoting or inhibiting tumor growth [3–6]. Therefore, research on the association between circRNAs and cancer assumes immense significance as it holds the potential to identify potential therapeutic targets and biomarkers for cancer, and conducting systematic gene drug development.

Drug research is crucial to cancer treatment, but it is expensive and lengthy process. It takes about 10–15 years for a new drug to be discovered and applied clinically, costing between 0.8–1.5 billion dollars [7–9]. Given these challenges, finding new indications from approved or established clinical drugs has emerged as an effective strategy, a process called drug repositioning, which can be achieved by identifying interactions between drugs and cancer [10–13]. Computational prediction of circRNA-cancer and drug-cancer associations is crucial for identifying potential RNA targets and candidate drugs that can guide subsequent wet-lab experiments, thereby advancing cancer therapy.

Many computational models have been proposed to address the tasks of circRNA-disease and drug-disease associations. These approaches can be roughly classified as network-centric methods and machine learning-driven methods. For the former, a heterogeneous network is constructed utilizing the relationships among different biomolecules. Subsequently, specific algorithms are employed to forecast potential associations by leveraging the information encoded within this network. For example, KATZHCDA [14] utilizes KATZ measure to identify disease-associated circRNA within the heterogeneous network that are integrated using disease-disease similarities, circRNA-circRNA similarities and circRNA-disease associations. CD-LNLP [15] adopted a linear neighborhood propagating labels strategy to identify the latent disease-associated circRNA. RWR [16] is a circRNA-disease association predictor utilizing restarted random walking method. BNNR [17] recovers the missing associations of the heterogeneous drug–disease network based on bounded nuclear norm regularization method. Xie et al. integrated the weighted K nearest known neighbors and bipartite graph diffusion to identify novel drug-disease associations [18]. However, most network-centric methods are unable to make association predictions for nodes without any interaction information. Machine learning-driven methods primarily utilize supervised or unsupervised learning approaches to mine deep features of the data and iteratively optimize model parameters to accurately predict potential associations. Niu et al. incorporates the Markov model into graph neural network to infer potential disease-associated circRNAs [19]. DMFCDA [20] and NMF-DR [21] are two matrix factorization-based models that predict disease-associated circRNAs and drugs, respectively. LAGCN [22] and HNRD [23] are two predictors that utilize neural networks to extract drug-disease features, incorporating attention mechanisms and neighbor information to enhance information extraction. Despite the promising results obtained by previous methods, most of them only consider node features, and combine them in a simplistic concatenate manner without explicitly modeling the complex information contained in the links between nodes. Their neglect of the importance of edge embeddings learning limits the ability to fully capture valuable information in network topology. Moreover, most prior methods tackle circRNA-disease and drug-disease tasks separately, lacking a systematic perspective to analyze their interactions and consequently overlooking the constraints and coordination among multiple biomolecules.

Here, we present AutoEdge-CCP, a novel model that systematically predicts cancer-associated circRNAs and drugs by explicitly learning edge embedding. Firstly, we integrate the data of circRNA-cancer, drug-cancer, and circRNA-drug associations to generate a multi-source heterogeneous network and extract similarity attribute features based on the nodes in the network. Next, the autoGNN with Explicit Link Information is employed to learn edge feature representations in the multi-source heterogeneous network through the message passing and readout phases. It introduces diverse intra-layer and inter-layer dimensions in the message passing neural network and utilizes a robust search algorithm to ensure the effectiveness of the searched Graph Neural Network (GNN) framework. Finally, AutoEdge-CCP leverages a learning-to-rank (LTR) framework to tackle the prediction of circRNA-cancer and drug-cancer associations as ranking problems. By constructing ranked lists of associated cancers for each query circRNA or drug, we facilitate more efficient analysis. Moreover, experimental results across multiple scenarios demonstrate the superiority of AutoEdge-CCP compared to other state-of-the-art methods. Furthermore, case studies validate the ability of AutoEdge-CCP to detect potential circRNA-cancer and drug-cancer associations.

## Results

### Datasets

Three types of nodes and three types of associations were collected from public databases to construct the heterogeneous network for predicting cancer-associated circRNAs or drugs. We retrieved circRNA-cancer associations from the circR2Cancer database, a meticulously curated resource with experimentally validated circRNA-cancer links. For drug-disease associations, we obtained data from the CTD database, which includes both curated and inferred associations, sourced from published literature and curated drug-gene interactions, respectively. Following previous studies[24], the circRNA-drug sensitivity data was obtained from the circRic database. We determined significant connections between circRNA and drug sensitivity using a Wilcoxon test, establishing an association when FDR < 0.05, by analyzing the correlation between circRNA expression and drug sensitivity. We excluded isolated nodes and focused solely on those nodes that have at least one edge in the multi-source heterogeneous network. As a result, we collected a total of 614 circRNA-cancer associations, 1197 circRNA-drug associations, and 523 drug-cancer associations, covering 407 circRNAs, 24 drugs, and 46 cancers, respectively. For the tasks related to cancer-associated circRNAs and drug prediction, we constructed two imbalanced datasets, denoted as *S*_1_ and *S*_2_, respectively. These datasets encompassed experimentally validated circRNA-cancer associations and drug-cancer associations as positive samples, while their corresponding unobserved pairs were considered as negative samples. Detailed statistical information for both datasets and their application in circRNAs-cancer and drug-cancer association tasks is shown in **Table 1**.

**Table 1:** Statistical information of the datasets. “#” represents the number.

Datasets	#CircRNA	#Drug	#Cancer	#Positive	#Negative
*S*_1_	407	-	46	614	18108
*S*_2_	-	24	46	523	581

### Experimental setup for multi-scenario application

In this study, multi-scenario applications of AutoEdge-CCP algorithm can be divided into two categories. In Scenario 1, our goal is to predict newly discovered circRNAs and drugs associated with cancer. These novel entities have entirely unknown connections with the candidate set of cancers, labeled as "associated cancer ranking for novel queries". In Scenario 2, our goal is to predict the missing associations between known circRNAs (or drugs) and candidate cancers, termed "associated cancer ranking for known queries".

**Fig 1:**
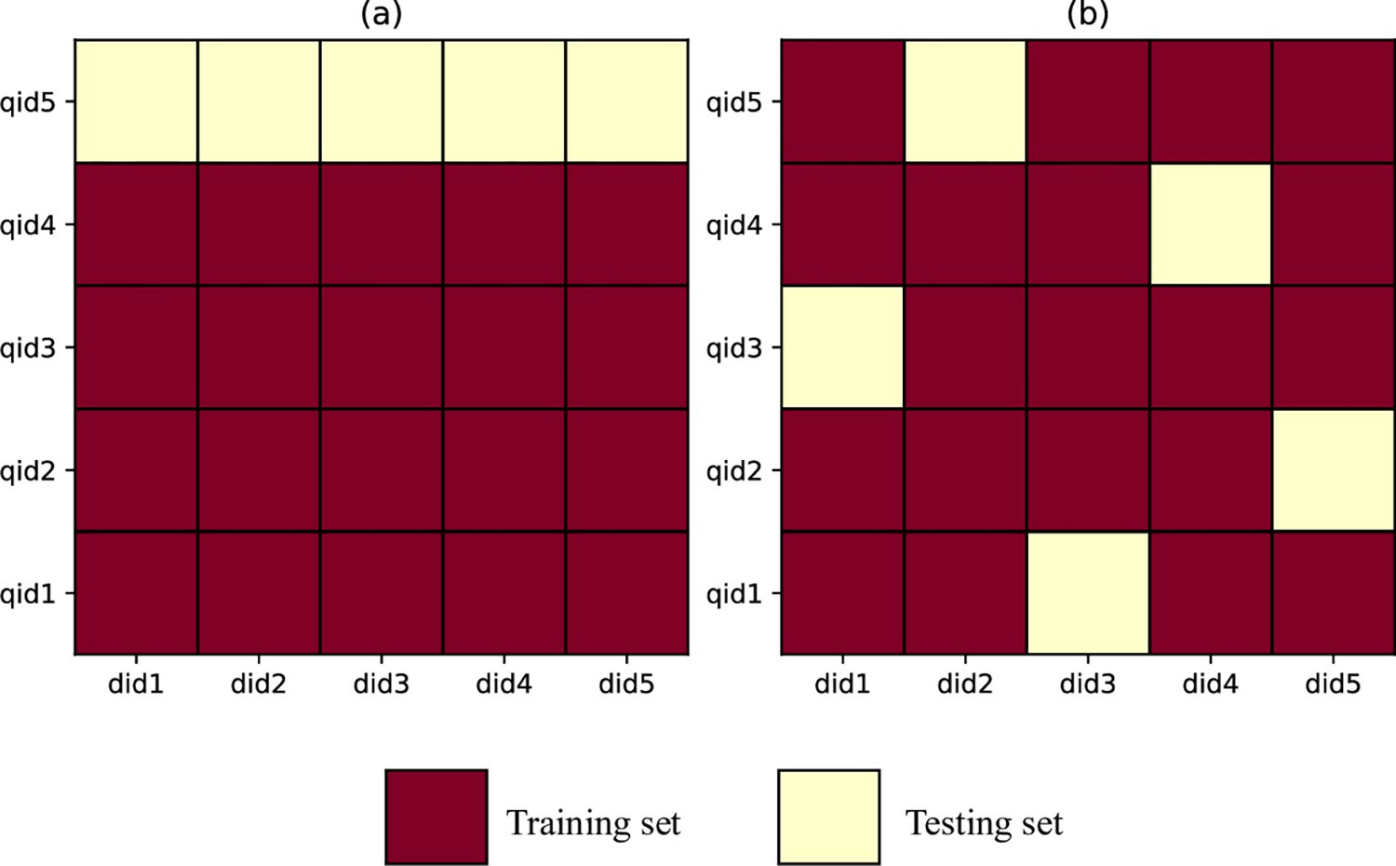
Distribution of datasets in two application scenarios. (a) Scenario1: associated cancer ranking for novel queries (b) Scenario2: associated cancer ranking for known queries.

For the first application scenario “associated cancer ranking for novel queries”, the distribution of dataset is shown in **Fig 1A**. There is no intersection of query ids between the training set and the test set. Specifically, the experimental process is conducted using a five-fold cross-validation approach. We assume the entire dataset comprises five circRNAs or drugs serving as queries, with their corresponding query ids labeled as qid1 to qid5. Using **Fig 1A** as an illustration, we divided the dataset into five non-overlapping subsets, each corresponding to a unique query id. We selected the subset corresponding to qid 5 as the test set, and remaining four subsets as the training set. This process is repeated five times, with the hold-out test set being changed to a different subset in each trial. Subsequently, the performance measures obtained from the five experimental runs were averaged to yield the final performance evaluation of the model.

For the second application scenario “associated cancer ranking for known queries”, the distribution of dataset is shown in **Fig 1B**. Partial data with each query is composed into a test set and the remain into a training set. During data split, all the dataset is randomly divided into five subsets. Similarly, the final experimental results are obtained using five-fold cross-validation.

### Parameter analysis

In order to comprehensively assess the performance and robustness of our proposed method, we conducted an in-depth parameter analysis. By systematically exploring the influence of various rankers and their key parameters on the results, we aimed to elucidate the optimal parameter configurations that yield the most accurate and reliable predictions. The detailed parameter settings of our implementation are provided in **S1 Table**.

To gain deeper insights into the impact of different rankers on the performance of the ranking model for ranking cancer list to circRNA queries, we compared the parameters of rankers 0–7, where each ranker represents a different algorithm: 0 (MART), 1 (RankNet), 2 (RankBoost), 3 (AdaRank), 4 (Coordinate Ascent), 6 (LambdaMART), and 7 (ListNet). As shown in **Table 2**, the results demonstrated that the LambdaMART model significantly outperforms the other models in terms of AUC and NDCG@10 matrics, indicating its suitability for the query associated cancer ranking tasks.

**Table 2:** Comparison of different rankers in LTR.

Ranker	AUC	NDCG@10
0: MART	0.9889	0.9524
1: RankNet	0.1438	0.5968
2: RankBoost	0.6757	0.9184
3: AdaRank	0.3176	0.7374
4: Coordinate Ascent	0.1299	0.8236
6: LambdaMART	0.9892	0.9568
7: ListNet	0.3136	0.7807

The primary parameters of the LambdaMART algorithm include the Number of Trees, Learning Rate, Number of Threshold Candidates, and Minimum Leaf Support. We leverage the larger S1 dataset, containing more samples and queries than S2 dataset, to optimize these parameters. By analyzing changes in the performance of AutoEdge-CCP on the S1 dataset, we can fine-tune the aforementioned parameters to achieve an optimal combination. Moreover, this study followed the principle of controlling variables, where other parameters were held constant at their default values while evaluating a particular parameter. The final performance results were obtained by averaging the performance scores from a five-fold cross-validation.

**Fig 2:**
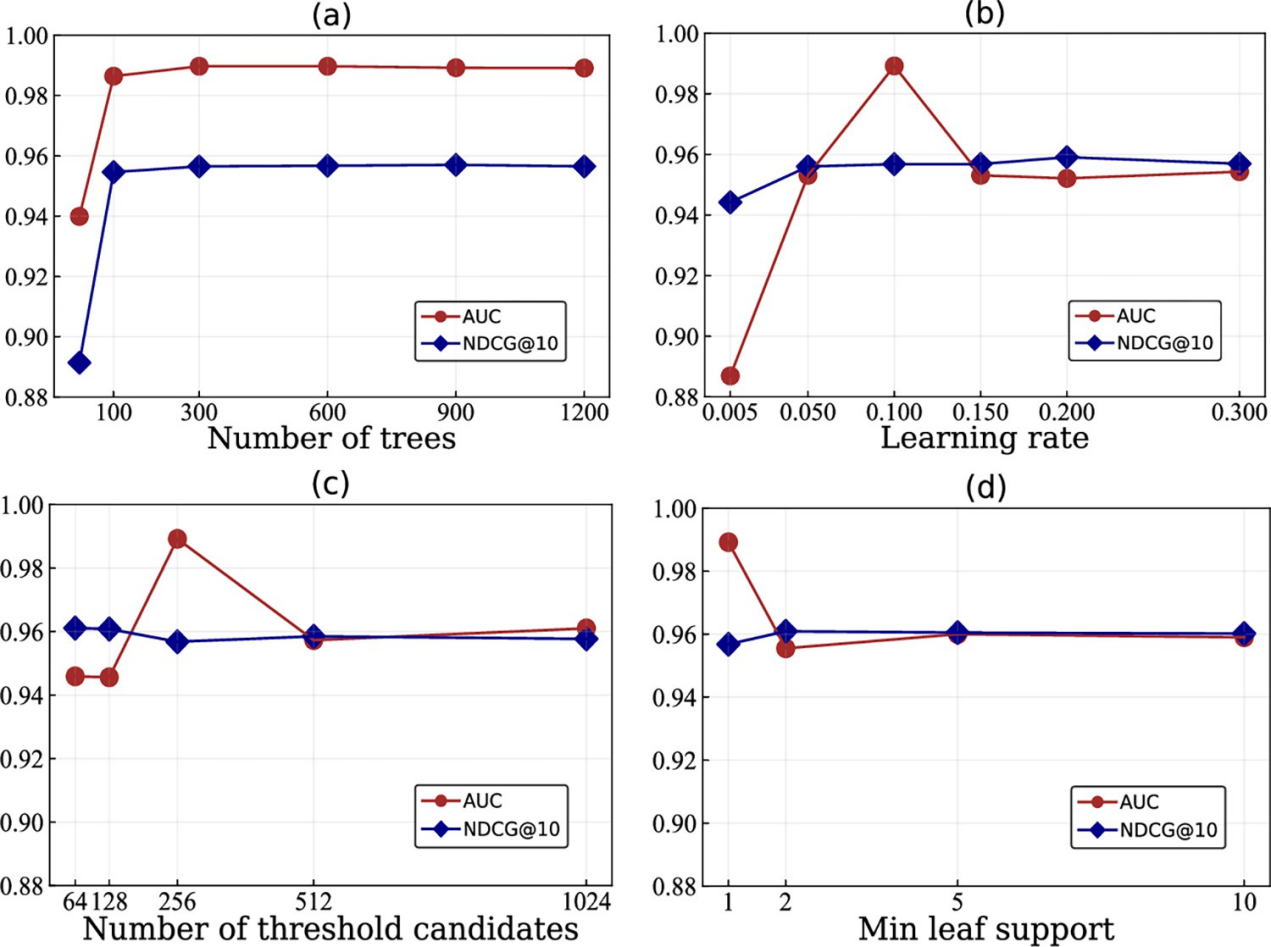
The impact of parameters of LambdaMART model. (a), (b), (c), and (d) respectively represent the AUC and NDCG@10 values obtained by AutoEdge-CCP under variations in the Number of Trees, Learning Rate, Number of Threshold Candidates, and Minimum Leaf Support.

The impact of parameter fine-tuning on the performance of the AutoEdge-CCP method is demonstrated in **Fig 2**. Notably, both the AUC and NDCG@10 metrics surpass 0.88, indicating the effectiveness of the LambdaMART algorithm in sorting cancer-related lists. Following a thorough comparison, we set the parameters of Number of Trees, Learning Rate, Number of Threshold Candidates, and Minimum Leaf Support to 1000, 0.1, 256, and 1, respectively. Other parameters, such as Number of leaves and estop, which have minimal impact on the model performance are set to their default values. With this combination, the AutoEdge-CCP method achieves better performance and generalization.

### Performance of AutoEdge-CCP in multiple scenarios

In Scenario1 of predicting associated cancer ranking for novel queries, we compared AutoEdge-CCP with five methods for circRNA-disease association prediction, including three machine learning-based methods, KATZHCDA [14], RWR [16], CDLNLP [15], and two deep learning-based methods, DMFCDA [20] and GMNN2CD [19] (**Table 3**). In addition, AutoEdge-CCP was compared with five drug-disease association prediction methods, including three machine learning-based methods, BNNR [17], NMFDR [21], BGMSDDA [18], and two deep learning-based methods, LAGCN [22], HNRD [23] (**Table 4**).

**Table 3:** Performance comparison of AutoEdge-CCP and other methods in novel circRNA associated cancers prediction.

Methods	AUC	AUPR	NDCG@10	NDCG	MRR	MAP
KATZHCDA	0.962	0.538	0.153	0.322	0.169	0.159
RWR	0.983	0.330	0.210	0.369	0.231	0.221
CDLNLP	0.482	0.018	0.154	0.450	0.324	0.154
DMFCDA	0.511	0.019	0.307	0.573	0.437	0.274
GMNN2CD	0.986	0.879	0.864	0.893	0.880	0.870
AutoEdge-CCP	**0.989**	**0.952**	**0.956**	**0.962**	**0.962**	**0.952**

**Table 4:** Performance comparison of AutoEdge-CCP and other methods in novel drug associated cancers prediction.

Methods	AUC	AUPR	NDCG@10	NDCG	MRR	MAP
BNNR	0.520	0.485	0.563	0.760	0.601	0.559
NMFDR	0.508	0.500	0.538	0.723	0.556	0.514
BGMSDDA	0.527	0.537	0.512	0.737	0.575	0.533
LAGCN	0.513	0.481	0.503	0.725	0.565	0.523
HNRD	0.481	0.467	0.463	0.563	**0.800**	0.466
AutoEdge-CCP	**0.700**	**0.665**	**0.699**	**0.794**	0.657	**0.652**

From the comparisons we can see that: (1) AutoEdge-CCP achieves the best comprehensive predictive performance in Scenario1, and obtaining a high-quality ranked list of associated cancers. (2) AutoEdge-CCP exhibits superior performance in predicting circRNA-associated cancer task within S1 dataset compared to the task of predicting drug-associated cancers in S2 dataset. This is consistent to the fact that AutoEdge-CCP, which is based on deep learning for feature extraction, exhibits good scalability and adaptability on large datasets. As a result, it can effectively utilize the information within the dataset to enhance the model’s generalization ability.

**Fig 3:**
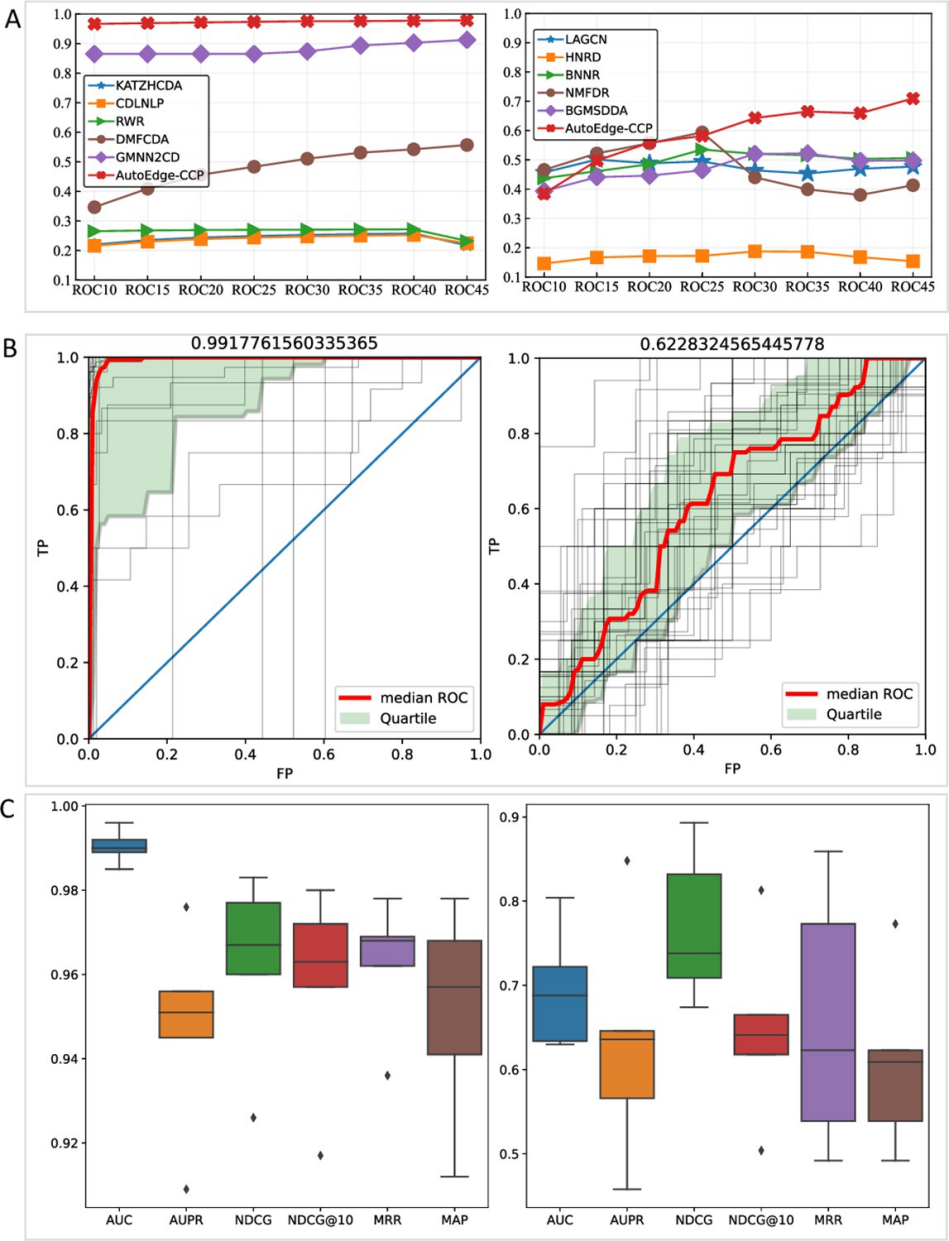
Performance of AutoEdge-CCP in multiple scenarios. (A) ROCk values comparison between AutoEdge-CCP and alternative methods in Scenario1. (B) Overall ROCs for 46 cancers. Median AUROC was shown on the top of each panel. Here, each gray line represents one cancer, the red line represents the median curve, and the light green part represents the region between the 25th and 75th quantiles. (C) Box plot depicting the metric scores of AutoEdge-CCP in Scenario 2. (A-C): left side presents circRNA-cancer association prediction, right side presents drug-cancer association prediction.

We compared the ROCk values of different methods with a specific range (ROC10-45) in Scenario1, as shown in **Fig 3A**. Given that our scenario is similar to information retrieval, it’s often most worthwhile to pay attention to the top k recommended results. The ROCk metric is precisely utilized to evaluate the ability of ranking top items. The area under the ROC curve can be extended to the metric of ROCk, that is the AUC for top k items. The formula for this metric is detailed in **S2 Text**. We can observe that AutoEdge-CCP is superior to all the competing methods for cancer-associated circRNA predicting. For drug-cancer associations, although some methods had higher ROCk values in the small range of k, AutoEdge-CCP outperformed other methods in the range of ROC25-45, indicating the advantages on large-scale datasets. Additionally, some methods show fluctuations or decreases, which can be explained by the uneven sorting ability of the model that leads to misjudgments of some samples.

**Fig 3B** demonstrates an extension of Scenario 1, presenting overall ROC curves from the perspective of 46 queried cancer types. The median values obtained for the circRNA-cancer and drug-cancer prediction tasks are 0.9917 and 0.6228, respectively.

To evaluate the performance of AutoEdge-CCP in multiple scenarios, we additionally applied it to predict associated cancer with known circRNAs or drugs in Scenario2. **Fig 3C** illustrates the results of the 5-fold experiments, demonstrating overall high accuracy and ranking capabilities in both known circRNAs (or drugs)-associated cancers.

### Evaluations of edge features derived from autoGNN

**Fig 4:**
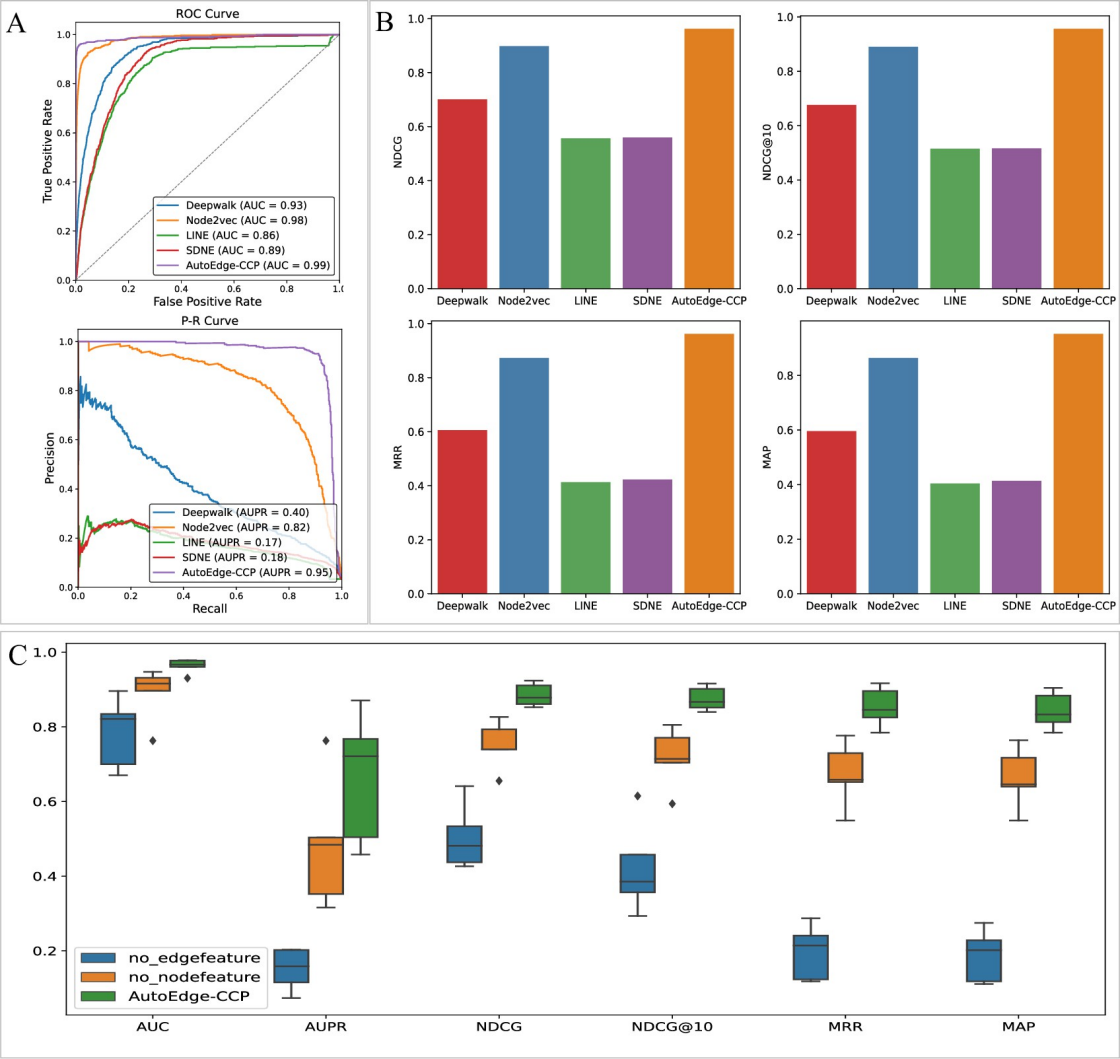
Analysis of the edge features derived from autoGNN. (A)-(B) Performance comparison under different graph embedding algorithms. (C) Performance comparison between AutoEdge-CCP and models without node feature or edge feature.

To assess the influence of autoGNN model on AutoEdge-CCP, we compare it with four classic graph embedding algorithms, including DeepWalk [25], node2vec [26], LINE [27], and SDNE [28], as shown in **Fig 4.** This experiment specifically focused on the circRNA-associated cancers task within Scenario1, while keeping the rest of the AutoEdge-CCP algorithm unchanged except embeddings model. The compared algorithms utilized default parameter settings.

As shown in **Fig 4A and 4B**, although other algorithms perform reasonably well on this scenario, their performance still falls short compared to AutoGNN. Specifically, we observed that AutoEdge-CCP achieved highest overall performance, improving the best-performing baseline, Node2vec, in terms of AUC, AUPR, NDCD, NDCD@10, MRR, and MAP by 0.6%, 13%, 6.4%, 6.6%, 8.9%, and 8.8%, respectively. These results suggest that autoGNN is better suited to mine the deep information contained in the associated data, improving the predictive performance of the AutoEdge-CCP algorithm for cancer association tasks in multiple scenarios.

In addition, we conducted ablation analysis by removing node features or edge features. As illustrated in **Fig 4C**, the results demonstrate that the model performs poorly when lacking node or edge features, highlighting their indispensability. Additionally, a greater improvement in performance with the incorporation of edge features, highlighting the effectiveness of autoGNN. To further explore the models’ robustness, we conducted isolated feature engineering on the three models to extract node GIP attribute features, mitigating potential data leakage. It is evident that AutoEdge-CC’s performance, despite a modest decline, remains commendable.

Moreover, we illustrated those parameters searched by AutoEdge-CCP and the ablated model, namely ‘no_nodefeature’, in **Table 5**. The AutoEdge-CCP model and the ablated model are adaptive to different graph neural network architectures. For combining operation, while the ablated model searched both sum operation for two layers, AutoEdge-CCP model adapted two concatenate operations. For activation operation, the ablated model searched Relu, Prelu functions in 1st layer and 2nd layer, respectively, while AutoEdge-CCP model selected reverse activation function order. For interlayer aggregation, the ablated model adapted none operation while AutoEdge-CCP concatenated two layers. Through the above analysis, it can be proved that AutoEdge-CCP can search the operation space to compose different graph neural network architectures.

**Table 5:** Two adaptive GNN framework for autoGNN and the ablated model.

Operations	AutoEdge-CCP	no_nodefeature
Agg	Max, Max	Sum, Max
Combine	Concat, Concat	Sum, Sum
Activation	Prelu, Relu	Relu, Prelu
Layer Connect	Skip_sum, stack	Stack, Stack
Layer Aggregation	Concat	None
Pool	Max	Max

### Visual explanations for AutoEdge-CCP

**Fig 5:**
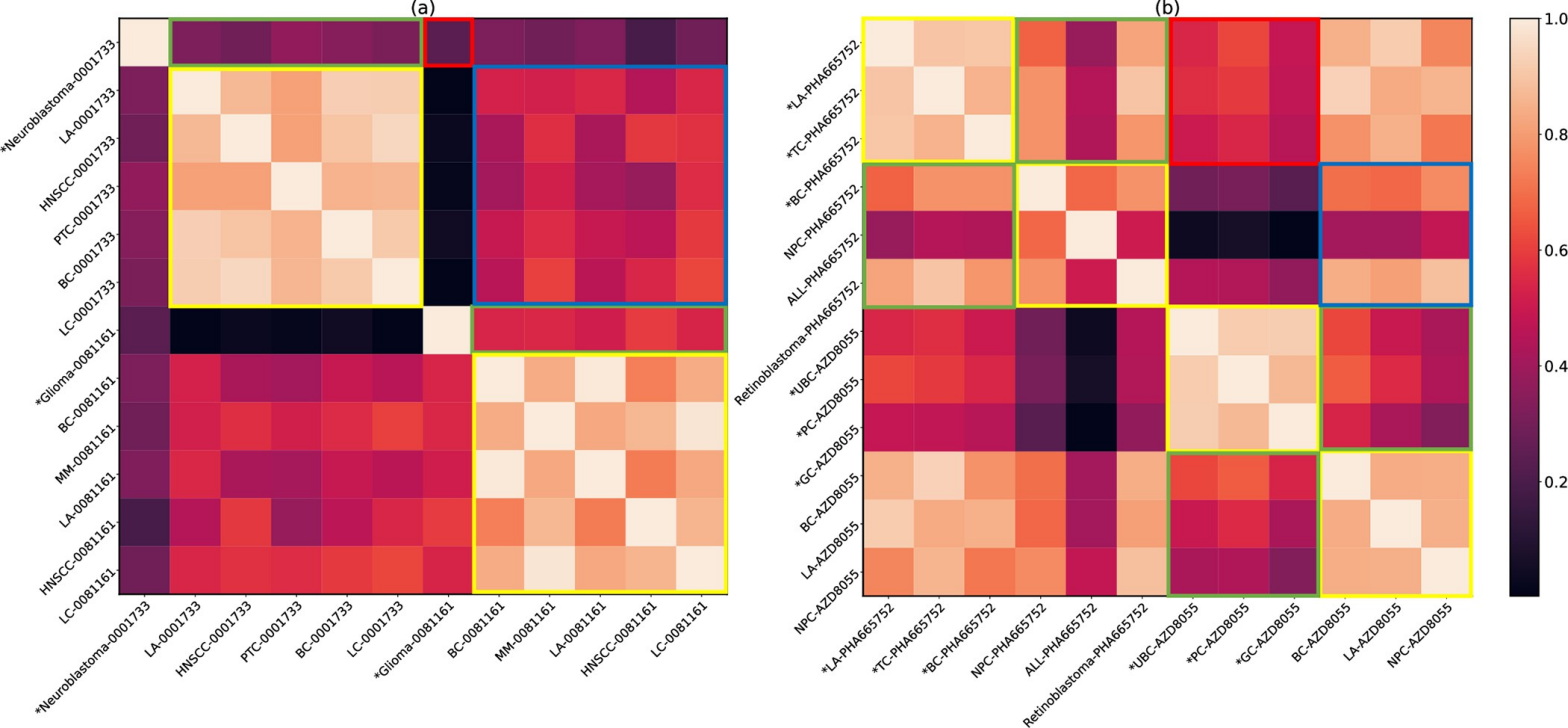
Heat maps of the similarity matrix for edge embedding. (a) and (b) represent the edge embedding similarity matrices learned by AutoEdge-CCP for 12 pairs of circRNA-cancer and drug-cancer, respectively. **Note**: * designates the labeled pairs, and the rest are unlabeled pairs. The abbreviations correspond to the following full names: hsa_circ_0001733 (0001733), hsa_circ_0081161 (0081161), Lung Adenocarcinoma (LA); Head and Neck Squamous Cell Carcinoma (HNSCC), Papillary Thyroid Cancer (PTC), Breast Cancer (BC), Liver Cancer (LC), Multiple Myeloma (MM), Thyroid Cancer (TC), Nasopharyngeal Carcinoma (NPC), Acute Lymphoid Leukemia (ALL), Urinary Bladder Cancer (UBC), Prostatic Cancer (PC); Gastric Cancer (GC).

We conducted a visual interpretation experiment to validate the rationale behind AutoEdge-CCP and observe its effectiveness in learning edge embeddings (i.e, *H*_*e*_ in **Eq 5**). Our objective was to understand the differences in the learning edge embeddings and their relevance to predicted results for circRNA-cancer and drug-cancer pairs. To achieve this, we computed Pearson correlation coefficients between different edge embeddings for these pairs. In the visual experiment, we illustrated two circRNA-cancer pairs and randomly selected five unlabeled (unobserved) pairs for each circRNA-cancer pair, while keeping the circRNA constant for comparison. Similarly, we randomly chose two drugs, with each having three labeled drug-cancer pairs and three unlabeled pairs. In **Fig 5A**, we can observe the following findings: (1) For the same circRNA, the edge embeddings with the same label (highlighted in the yellow rectangle) exhibit higher similarity compared to those with different labels (highlighted in the green rectangle). (2) For unlabeled pairs, the edge embeddings of different circRNAs (highlighted in the blue rectangle) exhibit lower similarity compared to the edge embeddings of the same circRNA (highlighted in the green rectangle). Even the edge embeddings of labeled pairs for different circRNAs (highlighted in the red rectangle) exhibit lower similarity than the edge embeddings with different labels of the same circRNA (highlighted in the green rectangle). These findings demonstrate that AutoEdge-CCP effectively captures the inherent differences between positive and negative samples, as well as among different circRNAs, thereby significantly enhancing the model’s predictive capacity. **Fig 5B** showcases the similarity matrices of edge embeddings for drug-cancer pairs, confirming the similar conclusions drawn from **Fig 5A**. This further validates the generalization ability of AutoEdge-CCP in learning effective link information.

### Case study

To verify the capability of AutoEdge-CCP in prioritizing unknown associations, we carried out case studies on queried circRNA (circ-RAD23B) and queried drug (NVP-AUY922) in Scenario1.

For circRNA circ-RAD23B, as shown in **Table 6**, it can be observed that the top three candidate cancers (Esophageal cancer, Colorectal cancer, Non-Small Cell Lung Cancer) have been supported experimentally validated in recently published literature. In specifically, circ-RAD23B regulates PARP2 and AKT2 by sponging miR-5095 in esophageal cancer [29]. The inhibition of circRAD23B has been demonstrated to impede the advancement of colorectal cancer through the regulation of the miR-1205/TRIM44 axis [30]. Additionally, circ-RAD23B has been found to impede the progression of non-small cell lung cancer by modulating the miR-142-3p/MAP4K3 axis [31].

**Table 6:** Top-ranked candidate cancers related to circ-RAD23B predicted by AutoEdge-CCP.

CircRNA	Rank	Candidate Cancers	Evidences
circ-RAD23B	1	Esophageal cancer	31208717
	2	Colorectal cancer	33634427
	3	Non-Small Cell Lung Cancer	35106926
	4	Bladder Cancer	NA
	5	Esophageal Squamous Cell Carcinoma	NA

In **Table 7**, the AutoEdge-CCP analysis reveals the top five candidate cancers with the highest probability of association with the drug NVP-AUY922. Interestingly, the corresponding literature confirms four of these cancer types, namely gastric cancer, breast cancer, non-small cell lung cancer, and colorectal cancer. For instance, NVP-AUY922, a potent inhibitor of heat shock protein 90, has demonstrated significant activity against gastric cancer cells [32]. Based on similar mechanism of action, NVP-AUY922 also has a potential growth inhibition effect in breast cancer cell lines [33]. Additionally, in vitro studies have shown that NVP-AUY922 significantly impedes the growth of all 41 tested non-small cell lung cancer cell lines with IC50 < 100 nmol/L [34]. The combination of NVP-AUY922 and TRAIL improves therapeutic outcomes in Colorectal cancer patients [35]. In addition, the candidate cancer (esophageal Squamous Cell Carcinoma) ranked in the top 5 associated with NVP-AUY922 was recorded in the CTD database.

**Table 7:** Top-ranked candidate cancers related to NVP-AUY922 predicted by AutoEdge-CCP.

Drug	Rank	Candidate cancers	Evidences
NVP-AUY922	1	Gastric cancer	21453385
	2	Breast cancer	18430202
	3	Non-small cell lung cancer	23493311
	4	Colorectal cancer	25446253
	5	Esophageal Squamous Cell Carcinoma	CTD

**Fig 6:**
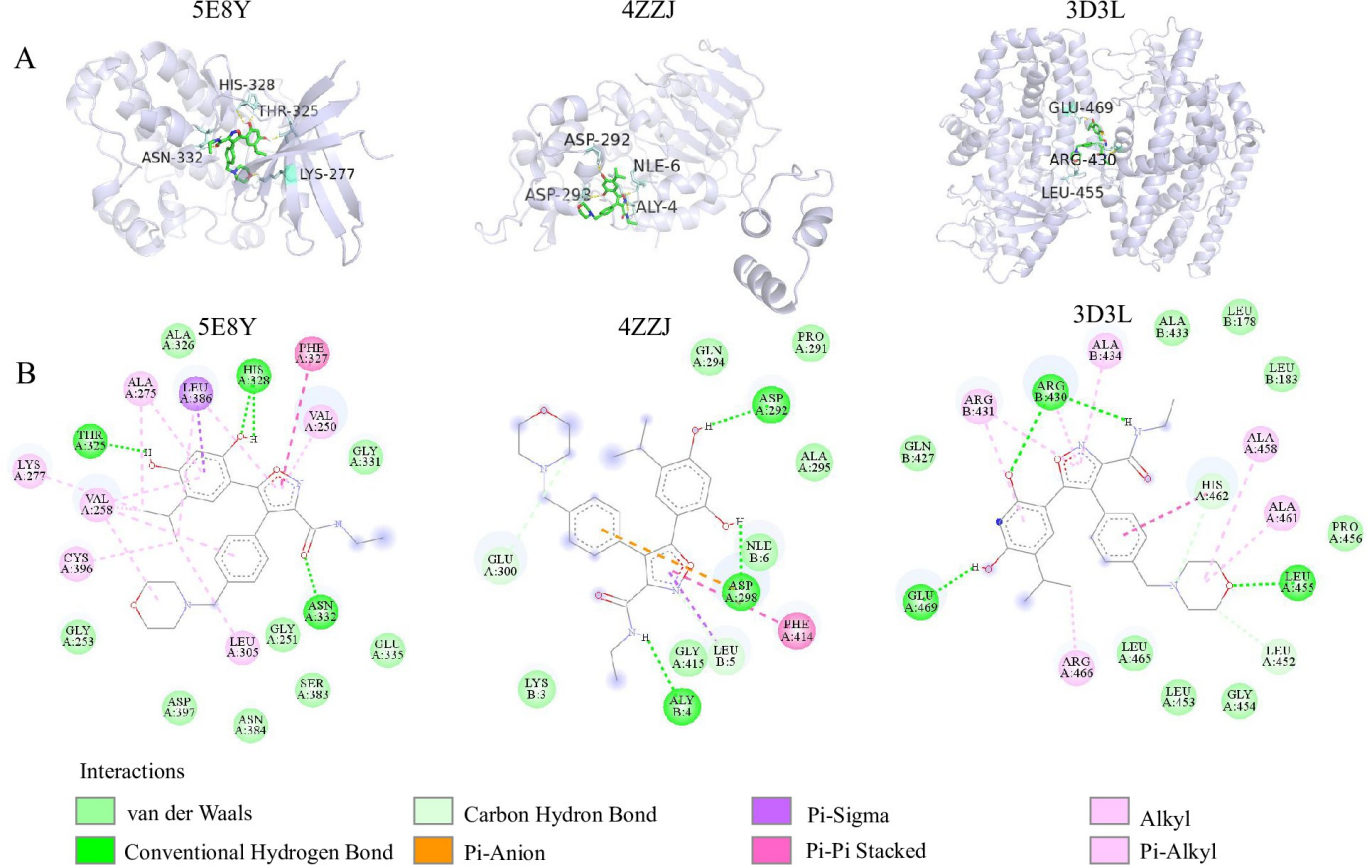
Visualization of NVP-AUY922 (PubChem CID: 135539077) and binding pockets. (A) The 3D representations of NVP-AUY922 with the binding pockets of 5E8Y,4ZZJ and 3D3L. (B) The interaction maps of NVP-AUY922 with 5E8Y,4ZZJ and 3D3L.

It is important to note that the CTD database source includes a combination of curated and inferred data, which might not hold the same level of authoritative validation. As a result, we intend to rigorously validate the predicted association through further investigation to ensure the reliability and accuracy of AutoEdge-CCP. We employed autoDockTools for molecular docking simulation experiments on the un-confirmed NVP-AUY922-Esophageal Squamous Cell Carcinoma association. The results were visualized using Pymol and DS software, as shown in **Fig 6**. We focused on three targets relevant to Esophageal Squamous Cell Carcinoma: TGF-beta receptor type-2 (TGFBR2) [36], Cellular tumor antigen p53 (TP53) [37], and Polyunsaturated fatty acid lipoxygenase (ALOX12) [38]. Human protein targets were selected from X-ray structures with resolutions above 2.5 Å, and their crystal structures (PDB IDs: 5E8Y, 4ZZJ, 3D3L) were retrieved from the Protein Data Bank (PDB) [39]. We obtained the docking binding energies of these targets with NVP-AUY922, represented by negative values where smaller negatives indicate higher efficacy. Additionally, we conducted molecular docking of NVP-AUY922 with three Colorectal Cancer targets, comparing the results with those for Esophageal Squamous Cell Carcinoma as outlined in **Table 8**. The results indicate that the molecular docking effectiveness of Esophageal Squamous Cell Carcinoma with NVP-AUY922 is comparable to the literature-supported interaction between Colorectal Cancer and NVP-AUY922. In the case of 5E8Y, as illustrated in **Fig 6B**, we have observed the presence of conventional hydrogen bond interactions between the compound and residues THR325, HIS328, and ASN332. Moreover, a range of hydrophobic interactions has been identified. These encompass residues like LYS277, CYS396, LEU305, VAL258, and ALA275 in alkyl interactions, LEU386 in PI-sigma interactions, PHE327 in pi-pi stacked interactions, and ALA275, LEU386, VAL250, and VAL258 in pi-stacked interactions. Additionally, Van der Waals interactions occur between other amino acid residues and the small molecule.

**Table 8:** The molecular binding energy of NVP-AUY922 with human target proteins associated with Esophageal Squamous Cell Carcinoma and Colorectal Cancer.

Cancer	Target			Binding energy (Kcal/mol)
	Protein	PBD ID	Reference	
Esophageal Squamous Cell Carcinoma	TGF-beta receptor type-2 (TGFBR2)	5E8Y	[36]	-7.06
	Cellular tumor antigen p53 (TP53)	4ZZJ	[37]	-5.85
	Polyunsaturated fatty acid lipoxygenase (ALOX12)	3D3L	[38]	-4.87
Colorectal cancer	Mothers against decapentaplegic homolog 4 (SMAD4)	1G88	[40]	-5.99
	Catenin beta-1 (CTNNB1)	1P22	[41]	-4.59
	DNA mismatch repair protein Mlh1 (MLH1)	6WBB	[42]	-4.47

## Discussion

We proposed AutoEdge-CCP, a novel method based on autoGNN with Explicit Link Information and LTR algorithm, to deal with the multi-association prediction of circRNA-cancer and drug-cancer. Compared with prior methods, AutoEdge-CCP offers the following advantages: (1) We combine isolated circRNA-cancer, drug-cancer, and drug-circRNA associations to create multi-source heterogeneous networks. These networks enable systematic integration analysis of circRNA-cancer and drug-cancer interactions, enhancing information complementarity. (2) AutoGNN explicitly models the edge feature engineering across both intra-layer and inter-layer dimensions of the message passing network, enabling comprehensive utilization of molecular interaction information for improved link prediction performance. (3) The use of an LTR algorithm transforms the association challenge into a ranking problem, allowing for a comprehensive assessment of candidate cancer relationships and reducing false positives, especially at the top level. Thus, AutoEdge-CCP is highly practical for predicting cancer associations with novel circRNAs and drugs. (4) The visualization of high-order edge embeddings and molecular docking experiments provides interpretable insights into the prediction outcomes, instead of black-box results.

In our future work, we can strive for additional advancements in our model through the following avenues. (1) Employing constrained design principles, guided by knowledge or rules, to enhance the intrinsic interpretability of the network structure (2) Delving into the diverse relationship types of circRNA-cancer and drug-cancer, encompassing facets such as promotion or inhibition, to facilitate more precise predictive capabilities.

## Materials and methods

### Problem formulation

In predicting cancer-associated circRNAs and drugs, the task is to train a model using a multi-source heterogeneous network as input, generating an output that discerns the absence of interactions between circRNAs (or drugs) and cancers. Specifically, the given heterogeneous network is defined as graph *G* = (*V*,*E*), where *v* includes circRNA sets *R* = {*r*_1_,*r*_2_,…,*r*_*m*_}, drug sets *D* = {*d*_1_,*d*_2_,…,*d*_*n*_}, and cancer sets *C* = {*c*_1_,*c*_2_,…,*c*_*k*_}, and E represents the edge sets. Our objective is to find a model M that maps the joint feature representations of nodes *c*_*k*_ and *r*_*m*_ (or nodes *c*_*k*_ and *d*_*n*_) to an interaction probability score pϵ[0,1].

### Overview of the AutoEdge-CCP framework

**Fig 7:**
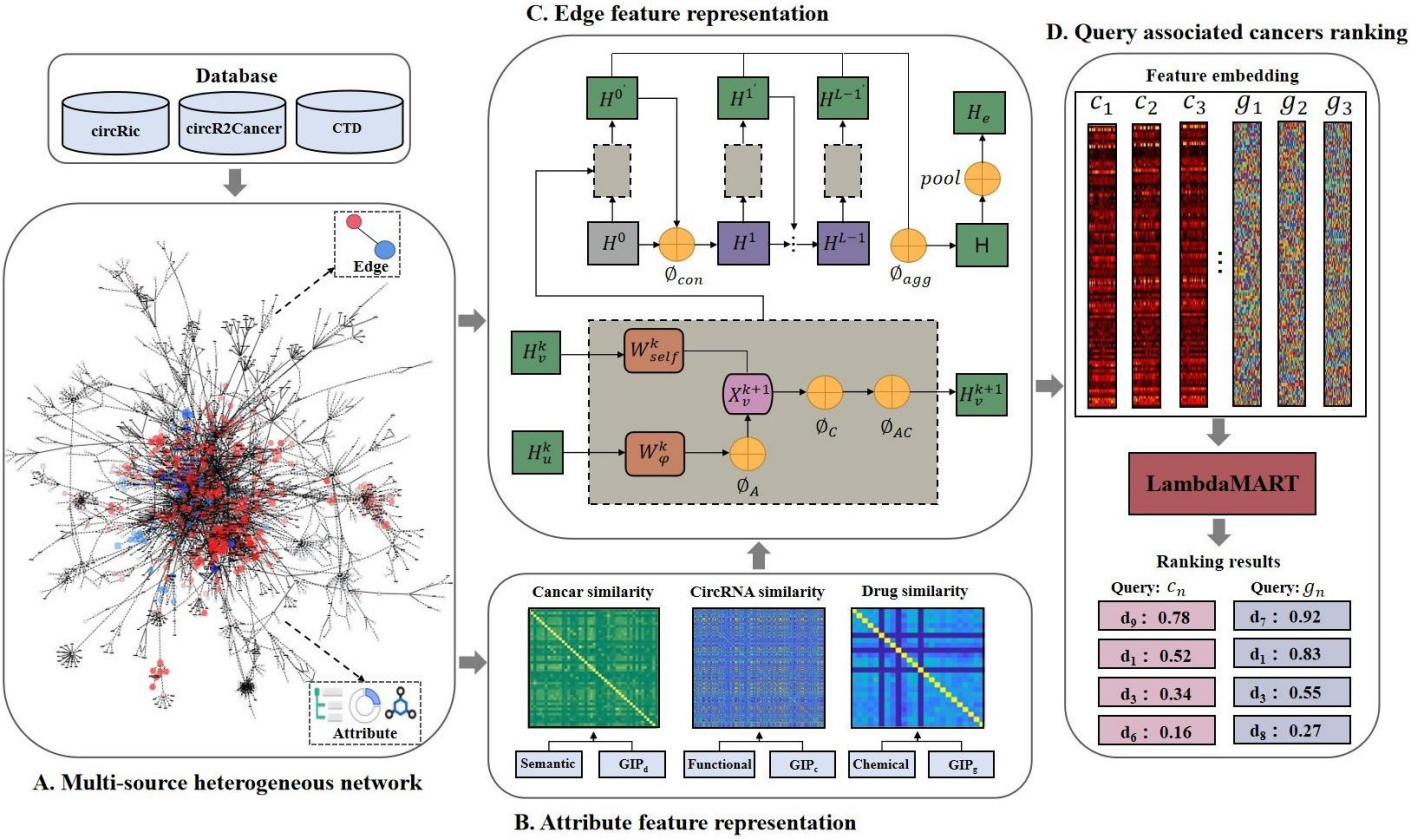
The framework of AutoEdge-CCP. There are four steps: (A). multi-source heterogeneous network construction. Integrating association data encompassing circRNA, drugs, and cancer from the circRic, circR2Cancer, and CTD databases. (B). Attribute feature representation. Extracting cancer, circRNA, and drug attribute features based on similarity calculations. (C). Edge feature representation. AutoGNN explicitly modeling link information to obtain edge features. (D). Query associated cancers ranking. The lambdaMART algorithm transforms the association problem into associated cancer lists ranking for queried circRNA or drug.

AutoEdge-CCP is proposed to deal with multitask: circRNA-cancer and drug-cancer association prediction. Our approach framework, as shown in **Fig 7**, consists of four steps: multi-source heterogeneous network construction, attribute feature representation, edge feature representation, and query associated cancers ranking. Details are provided in the subsequent sections.

### Multi-source heterogeneous network construction

In this study, we conceptualize biomolecules as nodes and interactions between molecules as edges, creating a multi-source heterogeneous network that effectively captures the intricate relationships among various biomolecules [43–45]. In the network, each node is represented by two types of information: intrinsic attributes information (such as circRNA functionality, drug compound structure, and cancer semantics) and edge information that captures the relationships between nodes. We collected three types of nodes (circRNA, drugs, and cancer) and diverse associated data, including circRNA-cancer associations, drug-cancer associations, and circRNA-drug sensitivity associations, from multiple public databases. After conducting a series of data processing operations, including deduplication, standardization of identifiers, and removal of non-human association data, we constructed a multi-source heterogeneous network consisting of 477 nodes and 2334 edges. This network enhances prediction of missing circRNA-cancer and drug-cancer associations from a systematic perspective by incorporating diverse information.

### Attribute feature representation

We calculate the cancer semantic similarity, circRNA functional similarity, and drugs chemical structure similarity. These features were then fused with GIP kernel similarity respectively to obtain attribute feature representations. The detailed calculation procedures are provided in **S1 Text**.

### Edge feature representation

In this part, our model employs AutoGNN with Explicit Link Information [46] algorithm to construct edge feature engineering of the multi-source heterogeneous network. The AutoGNN model can automate the appropriate GNN architecture design for the given data [47] and introduce edge embedding in an explicit way. The edge feature engineering consists of the message passing phase and readout phase.

### Message passing phase

Information is searched from the intra-layer message passing neural network (MPNNa) and inter-layer message passing neural network (MPNNr) during the message passing process. To encode the link information of the graph *G*, MPNNa utilizes a weak attention mechanism to differentiate between self-type and neighbor-type edges based on a linear transformation $W_{\varphi (u)}^{k}$, where *φ*(*u*)∈{*self*,*neigh*}. The MPNNa is instantiates as:

$$X_{v}^{k+1}=\emptyset_{A}(W_{\varphi (u)}^{k}H_{u}^{k}),\forall u\in N(v)$$ (1)

$$H_{v}^{k+1}=\emptyset_{AC}(\emptyset_{C}(W_{self}^{k}H_{v}^{k},X_{v}^{k+1}))$$ (2)

Where N(v) represents the neighboring nodes of *v*, $H_{u}^{k}$ and $H_{v}^{k}$ denote the hidden representation of the *u* and *v* from the last layer, respectively. ∅_*A*_ governs the message aggregation process from the neighborhoods of nodes. ∅_*AC*_(∙) defines the method of combining messages from a node’s own with those from its neighboring nodes. ∅_*C*_(∙) denote the activate function. The candidate choices for the above three operations are defined as: ∅_*A*_(∙)∈{*sum*,*max*,*mean*}, ∅_*AC*_(∙)∈{*sum*,*concat*}, and ∅_*C*_(∙)∈{*ReLU*,*PReLU*}.

Next, MPNNr acquires information across layers through both layer-wise connectivity and layer-wise aggregation. The layer-wise connectivity operation combines the output embedding *H*^*k*−1^ of the k-th MPNNa with the output embedding *H*^*k*^ of current layer to from a new representation *H*^*k*^, which is then fed into the subsequent layer. The layer-wise connectivity operation is defined as:

$$H^{k}=\left\{ \begin{array}{c} \emptyset_{con}(H^{k}),\emptyset_{con}=skip\\ \emptyset_{con}(H^{k-1}{,H}^{k}),\emptyset_{con}=sum\\ {W\emptyset }_{con}(H^{k-1}{,H}^{k}),\emptyset_{con}=concat \end{array}\right.$$ (3)

∅_*con*_(∙) denote the layer-wise connectivity function, where skip connectivity [48] in combination with two others helps alleviate the over-smoothing problem [49], and W is the linear transformation matrix. The layer-wise aggregation operation enables adaptive representation learning through layer-by-layer aggregating representations generated by each layer of MGNNa, which is defined as follows.

$$H=\left\{ \begin{array}{c} {\emptyset_{agg}(H}^{L}),\emptyset_{agg}=skip\\ {\emptyset_{agg}[H}^{1}\|\ldots \|H^{L}],\emptyset_{agg}=concat\\ {\emptyset_{agg}(H^{1},\ldots ,H}^{L}),\emptyset_{agg}=max \end{array}\right.$$ (4)

Where ∅_*agg*_(∙) represents the layer aggregation function.

### Readout phase

To obtain the final edge feature representation *H*_*e*_ from the set of nodes hidden embeddings in *G*, we introduce the powerful pooling operation *σ*(∙)∈{*max*,*concat*,*sum*}, which is expressed as follows:

$$H_{e}=\sigma (H_{v}|v\in G)$$ (5)

The autoGNN model employs the stochastic differentiable SNAS algorithm [50], rendering search objectives for multiple operations differentiable through reparameterization. This results in an efficient GNN framework achieved through adaptive searching. Assuming the search space *ε* for operations is sampled from the distribution *p*_*w*_(*ε*) parameterized by structured parameters *w*, it is defined as follows:

$$\varepsilon_{o}=\frac{exp((logw_{o}-log(-log(U_{o})))/\tau )}{\sum_{o^{\prime }\epsilon O}exp((logw_{o^{\prime }}-log(-log(U_{o^{\prime }})))/\tau )}$$ (6)

Where *o* signifies a candidate operation, *U*_*o*_~*Uniform*(0,1) represents uniform distribution sampling, and *τ* denotes the tolerance for the softmax activation function. This ensures that the probability of sampling *o* (i.e., *ε*_*o*_ = 1) is directly proportional to its weight *w*_*o*_. Moreover, the stochastic differentiable relaxation becomes unbiased upon convergence due to the one-hot characteristic with $\underset{\tau \to 0}{\lim}\;\varepsilon_{o}=1$. The search problem can be formulated as follows:

$$\underset{w,\theta }{\max}\;E_{\varepsilon ∼p_{w}(\varepsilon )}[f(\varepsilon ,\theta ;G)]$$ (7)

Where *f*(∙) denotes the performance of the designed AutoGNN model’s operation combination ε with weight *θ* on graph *G*, and *E*(∙) is the expectation.

### Query associated cancers ranking

LTR is a powerful technique that converts association problems into ranking problems in the domain of information retrieval [51]. Essentially, LTR enables us to retrieve and rank relevant documents from a candidate set based on a given query. The remarkable advantage of LTR lies in its ability to eliminate the need for constructing negative samples, making it highly suitable for handling data with imbalanced classes. Notably, LTR has demonstrated exceptional performance across various areas in bioinformatics, such as: prediction miRNA-disease identification [52], drug-target binding affinity prediction [53], protein structure and function [54], and protein remote homology detection [55].

The LTR algorithm can be classified into three categories—pointwise, pairwise, and listwise—distinguished by varying inputs and loss functions. The pointwise method focuses on the absolute relevance between individual documents and queries, the pairwise method assesses relative relevance by comparing the order of different documents, and the listwise method optimizes the entire sequence directly for ranking evaluation metrics. However, the primary focus of LTR is on sorting items rather than providing precise scoring outputs. Therefore, in this paper, we employ LTR to provide relative scoring results.

In this study, we adopted listwise type of LambdaMART to reframe the prediction tasks of circRNA-cancer and drug-cancer associations into circRNA or drug associated cancers ranking tasks for model training. This process parallels information retrieval. In topic-document retrieval, LambdaMART utilizes the joint features of each topic and its corresponding candidate document set as input. This algorithm then ranks the relevance of the candidate document set for a specific topic based on the degree of correlation. For circRNA or drug associated cancers ranking tasks, circRNAs or drugs serve as the queries, while multiple cancers serve as the candidates. LambdaMART’s goal is to prioritize associated cancers within the ranking list for each query. The open source toolkit of LambdaMART can be accessed within Ranklib (https://sourceforge.net/p/lemur/wiki/RankLib/).

The input and output data formats for this model are [*label*,*qid*,*features*] and [*qid*,*did*,*score*], respectively. In the input data, where each row represents a circRNA (or drug)-cancer pair sample, and the samples for the same query circRNA *i* (or drug *j*) have the same *qid*, the *label* indicates the correlation degree of circRNA (or drug)-cancer pair, when *label = 1*, it indicates that the sample has been experimentally verified to be associated; otherwise, *label = 0*, *features* are the edge features of circRNA (or drug)-cancer pairs, obtained by **Eq 5**. In the output data, where *did* is the unique id of the top cancer related to query *qid*, *score* denotes the predicted score of the corresponding circRNA (or drug)-cancer pair calculated by this model.

### Evaluation criteria

For the performance evaluation of AutoEdge-CCP, we employ a comprehensive set of measures for link prediction and ranking, including Receiver Operating Characteristic Curve (ROC) at k, the area under ROC (AUC), and Precision-Recall curve (AUPR), Normalized Discounted Cumulative Gain (NDCG), Mean Reciprocal Rank (MRR), and Mean Average Precision (MAP), details are provided in **S2 Text**.

## Supporting information

S1 Text The construction process of attribute feature representations for circRNA, cancer, and drug molecules. (PDF)

S2 Text Detailed descriptions of the evaluation metrics ROCk, NDCG, NDCG@K, MRR, and MAP. (PDF)

S1 Table List of value of hyperparameters in our model’s implementation. (PDF)
