# Supplementary material for: AutoEdge-CCP: A novel approach for predicting cancer-associated circRNAs and drugs based on automated edge embedding
Source: PLoS Comput Biol. 2024 Jan 30;20(1):e1011851. doi: 10.1371/journal.pcbi.1011851 (PMC10857569; doi:10.1371/journal.pcbi.1011851)
Supplement: S1 Text — (PDF) [file pcbi.1011851.s001.pdf]

**Attribute feature representation**

**Cancer-Cancer Similarity**

Cancer attribute feature representation is obtained by integrating the semantics and Gaussian Interaction Profile (GIP) kernel similarity of cancer. First, we calculate cancer semantic similarity according to Wang's strategy . According to the cancer ontology form stored in the Medical Subject Headings (MESH) database [1, 2], a given cancer $d$ can be represented by a Directed Acyclic Graph (DAG) $DAG_d = \{d, N_d, E_d\}$. Here, $N_d$ is the ancestor node set of cancer d, $E_d$ is the direct links among nodes. The semantic similarity between cancer $d_i$ and cancer $d_j$ is based on the nodes they share of DAG, as defined below.

$$SD(d_i, d_j) = \frac{\sum_{t \in N_{d_i} \cap N_{d_j}} (D_{d_i}(t) + D_{d_j}(t))}{\sum_{t \in N_{d_i}} D_{d_i}(t) + \sum_{t \in N_{d_j}} D_j(t)} \quad (1)$$

$$D_d(t) = \begin{cases} 1 & if\ t = d \\ max\{\mu \cdot D_d(t') | t' \in children\ of\ t\} & if\ t \neq d \end{cases} \quad (2)$$

Where $D_d(t)$ represent the semantic contribution value of cancer $t$ to cancer $d$ in $DAG_d$. $\mu = 0.5$ is the semantic contribution factor [3]. Since a directed acyclic graph cannot provide a complete coverage of all diseases in the MeSH database, we opted for the GIP similarity approach to obtain a more comprehensive measure of disease similarity. We calculate the GIP similarity of cancers using molecular landscape assumption that cancers with similar pathological characteristics are likely to be linked with similar circRNAs or drugs, and vice versa [4]. In the heterogeneous interaction network, there are three associations: circRNA-cancer, circRNA-drug, and drug-cancer. Therefore, we extend the GIP similarity model of each type of nodes based on two association matrices. Base on circRNA-cancer adjacent matrix, the GIP similarity $GD_1(d_i, d_j)$ between cancers $d_i$ and $d_j$ can be calculated using following formulations:

$$GD_1(d_i, d_j) = exp(-\theta_{d_1} \|V_1(d_i) - V_1(d_j)\|^2) \quad (3)$$

$$\theta_{d_1} = \frac{1}{n} \sum_{i=1}^{n} \|V_1(d_i)\|^2 \quad (4)$$

Where $V_1(d_i)$ represents the interaction profile used to encode the association of cancer $d_i$ with each circRNA, corresponding to the i-th column binary vector in circRNA-cancer adjacent matrix. $\theta_{c_1}$ denotes bandwidth parameter of GIP, and $n$ is the number of circRNAs. Base on drug-cancer adjacent matrix, the GIP similarity $GD_2(d_i, d_j)$ between cancers $d_i$ and $d_j$ can be calculated using following steps:

$$GD_2(d_i, d_j) = exp(-\theta_{d_2} \|V_2(d_i) - V_2(d_j)\|^2) \quad (5)$$

$$\theta_{d_2} = \frac{1}{m} \sum_{i=1}^{m} \|V_2(d_i)\|^2 \quad (6)$$

Where $V_2(d_i)$ and $\theta_{d_2}$ have the same meaning as $V_1(d_i)$ and $\theta_{d_1}$, $m$ is the number of drugs. Finally, to fully exploit the advantages of all available information, we obtain the cancer attribute feature representation as follows.

$$S(d_i, d_j) = \begin{cases} SD(d_i, d_j) & , if\ d_i, d_j\ has\ semantic\ similarity \\ (GD_1(d_i, d_j) + GD_2(d_i, d_j))/2 & otherwise \end{cases} \quad (7)$$

**CircRNA-CircRNA Similarity**

CircRNA attribute feature representation is accomplished by combining circRNA GIP and

functional similarity. We construct the circRNA function similarity model under the assumption if two circRNAs are shared by more semantically similar disease groups, the more functionally similar they are [5, 6]. Thus, the function similarity between circRNA $c_i$ and circRNA $c_j$ can be represented using following steps:

$$FC(c_i, c_j) = \frac{\sum_{d \in D_i} FD(d, D_j) + \sum_{d \in D_j} FD(d, D_i)}{|D_i| + |D_j|} \tag{8}$$

$$FD(d, D_t) = \max_{1 \le t \le n}(FD(d, d_t)) \tag{9}$$

Where $D_i$ and $D_j$ are the cancer sets related to circRNA $c_i$ and circRNA $c_j$, respectively. Based on the same principle as the cancer GIP similarity, we calculate the GIP similarity $GC_1(c_i, c_j)$ and $GC_2(c_i, c_j)$ of the circRNAs based on the circRNA-cancer and circRNA-drug adjacent matrices. Thus, the fusion circRNA attribute feature representation can be expressed as follows:

$$S(c_i, c_j) = \begin{cases} FC(c_i, c_j) & , \text{ if } c_i, c_j \text{ has function similarity} \\ (GC_1(c_i, c_j) + GC_2(c_i, c_j))/2 & \text{otherwise} \end{cases} \tag{10}$$

**Drug-Drug Similarity**

Drug attribute feature representation is obtained by combining drug chemical structures similarity and GIP similarity. The drug chemical structures similarity is determined using RDKit [7], a software package designed for cheminformatics and bioinformatics applications, based on SMILES (Canonical Simplified Molecular Input Line-Entry System) [8] of drugs. Firstly, the standard SMILES sequences of all drugs are retrieved from the DrugBank [9] Atlas, which are utilized to encode the chemical structure and spatial configuration of the drugs. The RDKit tool is then employed to transform the SMILES format into a numerical representation of drug fingerprints. Subsequently, the Dice coefficient [10] is used to measure the similarity between drug fingerprints, which is calculated by the following formula:

$$DG(g_i, g_j) = \frac{2|f_i \cap f_j|}{|f_i| + |f_j|} \tag{11}$$

where $|f_i|$ and $|f_j|$ represent the number of fingerprint bits of drug $g_i$ and $g_j$ respectively, and $|f_i \cap f_j|$ indicates the number of common fingerprint bits. Similar to circRNA, we construct the GIP similarity $GG_1(g_i, g_j)$ and $GG_2(g_i, g_j)$ of the drugs based on the circRNA-drug and drug-cancer adjacent matrices. Thus, we construct the drug attribute feature representation as follows:

$$S(g_i, g_j) = \begin{cases} DG(g_i, g_j) & , \text{ if } g_i, g_j \text{ has chemical structures similarity} \\ (GG_1(g_i, g_j) + GG_2(g_i, g_j)/2 & \text{otherwise} \end{cases} \tag{12}$$

**Reference**


1. Xiang Z, Qin T, Qin ZS, He Y. A genome-wide MeSH-based literature mining system predicts implicit gene-to-gene relationships and networks. BMC Systems Biology. 2013;7(3):S9.
2. Macintyre G, Yepes AJ, Ong CS, Verspoor K. Associating disease-related genetic variants in intergenic regions to the genes they impact. PEERJ. 2014;2:e639.
3. Wang D, Wang J, Lu M, Song F, Cui Q. Inferring the human microRNA functional similarity and functional network based on microRNA-associated diseases. Bioinformatics. 2010;26(13):1644-50.
4. van Laarhoven T, Nabuurs SB, Marchiori E. Gaussian interaction profile kernels for predicting drug–target interaction. Bioinformatics. 2011;27(21):3036-43.
5. Wang JZ, Du Z, Payattakool R, Yu PS, Chen C-F. A new method to measure the semantic similarity

of GO terms. Bioinformatics. 2007;23(10):1274-81.
6. Xiao Q, Luo J, Liang C, Cai J, Ding P. A graph regularized non-negative matrix factorization method for identifying microRNA-disease associations. Bioinformatics. 2018;34(2):239-48.
7. Bento AP, Hersey A, Félix E, Landrum G, Gaulton A, Atkinson F, et al. An open source chemical structure curation pipeline using RDKit. Journal of Cheminformatics. 2020;12(1):51.
8. Weininger D. SMILES, a chemical language and information system. 1. Introduction to methodology and encoding rules. J Chem Inf Comput Sci. 1988;28(1):31-6.
9. Wishart DS, Knox C, Guo AC, Shrivastava S, Hassanali M, Stothard P, et al. DrugBank: a comprehensive resource for in silico drug discovery and exploration. Nucleic Acids Research. 2006;34(suppl_1):D668-D72.
10. Dice LR. Measures of the amount of ecologic association between species. Ecology. 1945;26(3):297-302.
