# Supplementary material for: AutoEdge-CCP: A novel approach for predicting cancer-associated circRNAs and drugs based on automated edge embedding
Source: PLoS Comput Biol. 2024 Jan 30;20(1):e1011851. doi: 10.1371/journal.pcbi.1011851 (PMC10857569; doi:10.1371/journal.pcbi.1011851)
Supplement: S2 Text — (PDF) [file pcbi.1011851.s002.pdf]

Evaluation criteria

The ROC curve is a graphical representation that illustrates the relationship between the True Positive Rate (TPR) and the False Positive Rate (FPR). For query circRNAs or drugs, ROCk can be used to evaluate the sorting ability of ranking models when recommending the top k associated cancers, and its formula is as follows:

$$ROCk = \frac{1}{2}\sum_{i=1}^{k-1}(TPR_i + TRP_{i+1}) \cdot (FPR_{i+1} - FPR_i) \quad (1)$$

Where TPR_i and FPR_i represent the TPR and FPR of the first i cancers, respectively. AUPR measures the balance between precision and recall. Both AUC and AUPR have values from 0 and 1, and higher values suggest better performance of the model.

NDCG, MRR, and MAP comprehensively evaluate the ranking quality of models from different perspectives. NDCG captures the combined effect of relevance and ranking, MAP measures average precision across queries, and MRR considers the position of the first relevant item. For query circRNAs or drugs, NDCG, MRR, and MAP are formulated as:

$$NDCG@k = \frac{\sum_{i=1}^{k}\frac{2^{rel_i - 1}}{log_2(i+1)}}{\sum_{i-1}^{min(k,|R|)}\frac{1}{log_2(i+1)}} \quad (2)$$

$$MAP = \frac{1}{Q}\sum_{q=1}^{|Q|}\frac{1}{n}\sum_{k=1}^{n}P(k) \cdot rel_k \quad (3)$$

$$MRR = \frac{1}{Q}\sum_{q=1}^{|Q|}\frac{1}{rank_q} \quad (4)$$

Where NDCG@k is the NDCG evaluated for the top k associated cancers. rel_i is a binary variable that takes a value of 1 if the i-th ranked cancer is related to the query circRNA or drug, otherwise, it equals 0. $|R|$ represents the number of associated cancers, while n is the total number of cancers. Q is the number of the query circRNAs or drugs. $P(k)$ indicates the number of associated cancers within the top k candidates, and $rank_q$ refers to the ranking of the first associated cancer.
