# Supplementary material for: AutoEdge-CCP: A novel approach for predicting cancer-associated circRNAs and drugs based on automated edge embedding
Source: PLoS Comput Biol. 2024 Jan 30;20(1):e1011851. doi: 10.1371/journal.pcbi.1011851 (PMC10857569; doi:10.1371/journal.pcbi.1011851)
Supplement: S1 Table — (PDF) [file pcbi.1011851.s003.pdf]

Table 1. List of value of hyperparameters in our model's implementation. The parameters of autoGNN are tuned through hyperopt [1].

Parameters	autoGNN	Parameters	LambdaMART
Optimizer	Adam	Number of trees	1000
Learning rate	1e-4	Learning rate	0.1
Batch size	64	Number of threshold candidates	256
Search epoch	300	Min leaf support	1
MPNN layers	2	Number of leaves	10
Hidden dimension	100	Estop	100
Dropout	0	-	-

Reference

1. James B, Daniel Y, David C. Making a Science of Model Search: Hyperparameter Optimization in Hundreds of Dimensions for Vision Architectures. 2013/02/13: PMLR. p. 115-23.
